# Clinical Verifications

**Published:** 1888-06

**Authors:** H. C. Morrow


### CLINICAL VERIFICATIONS.

Mr. H. G. A. (grandson of General Gates, of Revolutionary fame), age fifty-six, came to me with dropsy of six months standing. Legs, feet, abdomen very much swollen, œdematous. Could scarcely put on his shoes, which were usually full large. Scrotum much swollen, watery appearance. Could not button waistband by three inches. I gave him empirically Apocynum can., on which he was kept in various doses and potencies for two weeks with only unsatisfactory results. I then concluded this was not Homœopathy, and took a careful statement of his symptoms, which I found to be as follows:

Gnawing hunger early in the morning before breakfast, relieved by eating. Feels full and bloated after eating. Loud eructations of tasteless gas for half an hour at a time, which relieve. Thirst for large quantities of water frequently. Rumbling of wind in the bowels. Diarrhœa of foul-smelling stools, rushing him out; can scarcely reach the water-closet. Seldom and scanty passage of high-colored urine, leaving red sediment in vessel which is difficult to wash out. It is unnecessary to say that Lycopodium was the remedy. March 16th, 1887, Lycop.$^{m}$ one dose a day. In one week he reported: Every vestige of dropsy was gone. Said he had been passing a gallon of water a day, had a good appetite, and felt better than for two years, and remains well at the present time. An allopathic physician offered to bet fifty dollars that he would die, and if I had not changed my prescription the bet would have been won. My first prescription was for the disease (diagnosed as Bright's by above mentioned allopath), the second for the patient.

Mrs. S., age twenty-seven, married, mother of children, quite fleshy. Has terrible paroxysms of cramping, aching, burning pains, commencing in right ovary and extending to the left side and lower part of abdomen (uterus); last from two to six hours. Not relieved by any position or application, but aggravated by hot (wet) applications. Great soreness all through pelvic cavity after attacks pass off. General symptoms: rumbling in abdomen; bloating, fullness in bowels not relieved by eructations, but better from emissions of flatus and rubbing. Pain in right ovary running down the limb. Urine looks like strong coffee, and at times blood red; *sensation in stomach as if everything were swinging back and forth.* The last symptom, which is very peculiar, I could find only under Lycopodium, and as it covered the remaining symptoms I gave one dose 71m dry on the tongue.

In about a week reported better. Above-mentioned symptoms and conditions all subsided. Had only one more attack of ovaritis and that was light. In about two weeks again reported, and here follows the key to the case: About a year ago she had an ulcer in the vagina and an excoriating leucorrhœa. The ulcer was cured (?) by the local application of Carbolic acid, and the leucorrhœa by daily injections of Hydrastis. Both the ulcer and the leucorrhœa disappeared, to be followed by the ovaritis and the cramps. I found now an ulcer on the left labium major externally. Patient says (I made no examination), ulcer two inches long, covered with dirty yellow pus, edges hard, sensation as of needles sticking in the edge, especially at night; beating and throbbing at night so she cannot sleep. Other symptoms: Leucorrhœa acrid, excoriating, profuse, thick, yellow, sometimes putrid, flows more when walking. Itching of pudendum by scratching. Pain in sacral region. Vertigo passing off by closing the eyes. Great desire for fat meat, pork especially, after eating which the ulcer pains more severely. Left hand "goes to sleep" when resting it quietly, and also night in bed. All these symptoms are found under Nitric acid, and Nitric acid$^{m}$ one dose cured the ulcer and the leucorrhœa.

H. C. MORROW.

